# Pathway thermodynamic analysis postulates change in glutamate metabolism as a key factor in modulating immune responses

**DOI:** 10.1097/IN9.0000000000000077

**Published:** 2026-02-25

**Authors:** Sunayana Malla, Rajib Saha

**Affiliations:** 1 Chemical and Biomolecular Engineering, University of Nebraska-Lincoln, Lincoln, NE, USA

**Keywords:** temperature, thermodynamic feasibility, driving force, immune activation, glutamate fate

## Abstract

**Background:**

Temperature, as seen during fever, plays a pivotal role in modulating immune responses and maintaining cellular homeostasis. Shifts in temperature influence the thermodynamic feasibility of metabolic reactions, with Gibbs free energy (ΔG) serving as a key indicator of the spontaneity of reactions under specific conditions. By altering ΔG in response to temperature changes across various metabolite concentrations and cell types, we can gain insights into the thermodynamic properties of metabolic pathways and identify critical factors involved in metabolism and immune function. Using Max-min Driving Force (MDF) analysis, we can assess changes in ΔG by varying temperature and metabolite concentrations, allowing for a detailed examination of thermodynamic feasibility at both the pathway and individual reaction levels.

**Method:**

Minimum driving force analysis was conducted to estimate the thermodynamic feasibility of metabolic pathways, including glycolysis, gluconeogenesis, oxidative phosphorylation, the pentose phosphate pathway, the tricarboxylic acid cycle, arginine and proline metabolism, amino sugar and nucleotide sugar metabolism (collectively referred to as amino sugar metabolism), leukotriene metabolism, and other amino acid pathways. The analysis was performed across a temperature range of 310.15 to 314.15 K. In addition, the ΔG for each reaction was calculated using standard Gibbs free energy values obtained from the equilibrator.

**Results:**

In this study, MDF analysis is applied to measure the changes in the driving force of pathways and the ΔG of each reaction at normal human core temperature (310.15 K) and elevated temperatures (up to 314.15 K). Additionally, we explore how shifts in the thermodynamic feasibility of reactions under immune activation, compared with normal physiological conditions, highlight key metabolic intermediates, such as fructose-1,6-bisphosphate, glucose-6-phosphate, and several steps in glutamate utilization, as important regulators of metabolic processes and immune responses.

**Conclusion:**

In conclusion, this study demonstrates that MDF-based thermodynamic analysis effectively captures temperature-dependent shifts in metabolic pathway feasibility and highlights glutamate metabolism as a key regulator of immune function. These findings underscore the utility of thermodynamic frameworks in advancing system-level understanding of human metabolism and immune regulation.

## 1. Introduction

Temperature fluctuations, such as fever (hyperthermia), have profound effects on the immune system ^[1]^ and cellular homeostasis. The immune system is susceptible to environmental changes, and temperature shifts can directly affect the metabolism, activation, and efficiency of several immune cells, such as macrophages, neutrophils, erythrocytes, and dendritic cells ^[2]^. At the molecular level, temperature alters the physical properties of cellular membranes, protein stability, and enzyme activity, which cascades into broader effects on immune cell metabolism, such as folding of proteins and expression of heat shock proteins ^[3]^. For a comprehensive understanding of these phenomena, thermodynamic analysis provides essential insights into the underlying physical chemistry that governs metabolic and immune processes. Reactions in immune cells must have sufficient driving forces, represented by the Gibbs free energy (∆G^o^/ΔG), to proceed efficiently ^[4]^. When temperature changes, the Gibbs free energy shifts, potentially making some reactions more or less feasible. Thermodynamic analysis helps elucidate how enzyme activity changes with temperature, affecting reaction rates within metabolic pathways ^[5]^. Several studies have explored the thermodynamic feasibility in immune cells, focusing on energetic demands, ion gradients across cell membranes, and changes in ΔG for antibodies that induce immune responses ^[3,6]^. However, pathway thermodynamics to decipher cell capabilities at different conditions, such as metabolite concentration changes and temperature fluctuations, have not been fully explored yet.

Metabolic pathways such as glycolysis, pentose phosphate (PPP), oxidative phosphorylation (OXPHOS), tricarboxylic acid (TCA) cycle, pyruvate metabolism, and several amino acid metabolism are fundamental to cellular energy production and have been increasingly recognized for their roles in modulating immune responses during infections and diseases ^[7–10]^. These pathways not only sustain Adenosine TriPhosphate (ATP) generation and biosynthetic needs under normal conditions but also undergo reprogramming to support heightened immune demands, such as rapid proliferation, cytokine secretion, and reactive oxygen species (ROS) production ^[11]^. However, the specific influence of fever—a common physiological response to infection that elevates body temperature on these pathways remains largely underexplored, with limited studies addressing its effects on individual enzymatic reactions or the integrated pathway dynamics ^[12]^. Recent advancements have highlighted the role of metabolism in shaping overall immune responses with different cell types, such as macrophages (and their activated phenotypes), neutrophils, mast cells, dendritic cells, T cells, and B cells, delineating heterogeneous metabolic behavior ^[13,14]^. For instance, pro-inflammatory M1 macrophages and effector T cells favor aerobic glycolysis (the Warburg effect), where glucose is rapidly converted to lactate even in oxygen-rich environments, providing quick ATP and intermediates for nucleotide and lipid synthesis ^[15,16]^. This is complemented by the PPP, which generates Nicotinamide Adenine Dinucleotide Phosphate for ROS production during oxidative bursts essential for pathogen killing and modulates macrophage polarization ^[15]^. OXPHOS, occurring in mitochondria, supports sustained energy in anti-inflammatory or memory cells via efficient ATP production from pyruvate oxidation ^[8]^. The TCA cycle integrates carbohydrate, fat, and amino acid metabolism, producing intermediates such as succinate and itaconate that act as signaling molecules to regulate inflammation—succinate stabilizes Hypoxia-Inducible Factor-1 Alpha for pro-inflammatory effects, while itaconate inhibits succinate dehydrogenase for anti-inflammatory resolution ^[17]^. However, the effect of temperature on each of these pathways individually is not fully explored. A 2024 study marks progress by isolating temperature effects on OXPHOS, revealing Enhancer of Caprice and Triptychome 1 as a vulnerable point, but it overlooks glycolysis, PPP, TCA, pyruvate, and other key metabolic pathways ^[18]^. Similarly, in conditions like dengue infection, metabolic shifts such as glycolytic upregulation are found to be significant, but the fevers’ contribution is not parsed. Also, physiologic disruptions in sepsis highlight systemic reprogramming, yet temperature-controlled experiments for such conditions are rare. Hence, temperature-controlled in-vitro studies could clarify these flux changes and potentially point to therapeutics—like Enhancer of Caprice and Triptychome 1 modulators—that reduce fever-induced metabolic burden without compromising immune benefits ^[7]^.

Taken together, these gaps underscore a broader limitation: although temperature profoundly shapes human immune function, fever is rarely incorporated into thermodynamic analyses of infection, and current applications of thermodynamic feasibility analysis remain focused mainly on simple model organisms like yeast and *Escherichia coli*, with few studies extending these approaches to human cells ^[5,19]^. Methods such as Thermodynamic-based Metabolic Flux Analysis (TMFA) integrate thermodynamic constraints into metabolic flux models to ensure that all reactions in a metabolic network are thermodynamically feasible ^[20]^. However, TMFA requires strict assumptions about metabolite concentrations and environmental conditions, which may not accurately reflect the actual dynamic conditions within cells, particularly in complex organisms ^[20,21]^. These fixed assumptions can make it challenging to capture the real flexibility and adaptability of the cellular metabolism, where metabolite concentrations and environmental factors often fluctuate ^[20,21]^.

On the other hand, the Max/min Driving Force (MDF) concept can identify which reactions become thermodynamically constrained or more favorable as temperatures fluctuate or physiological states change. This offers critical insights into metabolic robustness, efficiency, and adaptability, particularly for the immune cells such as neutrophils, macrophages, dendritic cells, and erythrocytes, which are often at the forefront of immune responses, including oxygen transport ^[1]^. By comparing these cell types, MDF can elucidate their metabolic adaptations, such as the high glycolytic flux in neutrophils during inflammation ^[22]^, more active lipid metabolism in macrophages ^[16]^, energy demands of dendritic cells during antigen presentation ^[23]^, and erythrocytes’ reliance on anaerobic metabolism ^[24]^. These insights provide valuable information on cell-specific vulnerabilities and pathways, informing therapeutic strategies for immune modulation and metabolic interventions in disease contexts. By focusing on maximizing the smallest driving force in a pathway, MDF can highlight reactions that become bottlenecks as temperature changes ^[19]^. This approach is particularly critical because it not only ensures overall pathway feasibility but also pinpoints specific steps that may need adjustment or optimization in response to temperature shifts and concentration changes. However, MDF is not without limitations: it often relies on a pre-specified flux distribution (eg, from Flux Balance Analysis) as input, which can introduce biases from the underlying flux prediction method compared with more integrated approaches such as TMFA. Additionally, standard MDF implementations assume enthalpy (ΔH) and entropy (ΔS) to be constant with temperature, neglecting temperature-dependent shifts in reaction thermodynamics; employ fixed metabolite concentration ranges (eg, 1–10 mM) that may not reflect cell-type-specific or dynamic physiological states; and omit kinetic or regulatory constraints such as enzyme saturation, allosteric control, or post-translational modifications. Despite these constraints, MDF’s flexibility in exploring variable concentrations and temperatures—without rigid stoichiometric or equilibrium assumptions—makes it particularly well-suited for studying dynamic shifts in reaction and pathway feasibility across cell types under thermal stress (eg, from 310.15 to 314.15 K), as in the present immune response analysis.

To this end, we applied the MDF analysis on pathways that are known to be an integral contributors in shaping immune responses namely glycolysis, gluconeogenesis, OXPHOS, PPP, TCA cycle, arginine/proline, amino sugar, and nucleotide sugar metabolism (mainly referred as amino sugar metabolism), leukotriene metabolism and other amino acid pathways, many of which were also identified by Ippolito et al ^[25]^ as the pathways that showed significant deviation to heat stress. We used metabolite concentrations expected in normal physiological conditions (0.01–10 mM) and further explored the pathway activity for highly essential immune cells, namely macrophages, neutrophils, erythrocytes, and dendritic cells, with an increase in temperature by constraining the metabolite concentrations as reported by Hooftman et al ^[26]^ and Kaiser et al ^[27]^, 2020. We identify the reactions that show a dynamic shift in thermodynamic feasibility from each of the pathways mentioned above when the temperature increases from normal body temperature (310.15 K) to fever conditions (312.15 K) to 313.15 K to 314.15 K, which can be dangerous and warrant immediate medical attention ^[1,28,29]^. The MDF analysis highlights the reactions associated with the utilization of glutamate to produce several factors, such as ornithine, citrulline, and G5SSH (l-glutamate-5-semialdehyde), as significant contributors in metabolic regulation and immune responses. Overall, the results of this study add to the importance of pathway and reaction-level analysis of temperature and the benefits of using thermodynamics to identify potential bottlenecks and trigger points.

## 2. Method

### 2.1 Standard Gibbs free energy

∆G^o^ (standard Gibbs free energy) was obtained for reactions originating from nine pathways using the equilibrator tool ^[30]^. Equilibrator is a tool that uses the composition contribution method to calculate the Gibbs free energy of formation at standard conditions ^[30]^. The list of reactions and the obtained standard ∆G^o^ values is available in Supplementary Table S1, https://links.lww.com/IN9/A7.

### 2.2 Thermodynamic modeling of metabolic pathways

Thermodynamic feasibility and pathway efficiency were analyzed using a matrix-based method integrating reaction stoichiometry with Gibbs free energy data, allowing systematic determination of feasible metabolite concentration ranges that ensure forward pathway flux as described in Noor et al ^[19]^. Each metabolic pathway was represented by a stoichiometric matrix S € R^m x n^, where m is the number of metabolites and n is the number of reactions. Each matrix element S_*ij*_ corresponds to the stoichiometric coefficient of metabolite *i* in reaction *j*, with negative values assigned to reactants and positive values to products ^[19]^. All reactions were formulated in the direction of their net physiological flux, and stoichiometries were based on the true molecularities of the enzymatic mechanisms rather than scaled or normalized coefficients. This convention ensures consistency and preserves the flux–force relationship applied in subsequent thermodynamic analyses. For our nine pathways, the S*ij* matrix was obtained from the global human Human1 model ^[31]^. The details on the calculation of MDF and change in ∆G can be found in Noor et al ^[19]^ and Chowdhury et al ^[32]^.

#### 2.2.1 Reaction and overall driving force

First, the driving force of each reaction is calculated from the standard Gibbs free energies and defined metabolite concentration ranges by using the following equation:


$$\Delta rG=\Delta rG{\;}^{\circ }+RT\cdot S^{T}\cdot x$$ (1)

where ∆G^o^ is the standard Gibbs free energy of each reaction, ∆G is the change in Gibbs free energy, R is the universal gas constant, T is the temperature in Kelvin, and x is the logarithm of metabolite concentrations in molar units. We varied the T from 310.15 to 314.15 K to obtain a change in ∆G values. To evaluate pathway thermodynamic feasibility, ∆G^o^ was treated as constant while varying x and T. A pathway was considered feasible if and only if at least one solution exists for the linear system defined by the constraints $\ln\left(C_{min}\right)\leq x\leq \ln\left(C_{max}\right)$ and ΔrG>0. In other words, there must exist a set of metabolite concentrations within the predefined range (in our case, 0.01–10 mM) such that all reactions exhibit a positive driving force. The concentration bounds can be specified uniformly across all metabolites or individually for each compound. The formulation of MDF can be found in Elad Noor et al ^[19]^. The pathway-specific driving force, along with $\Delta rG{\;}^{\circ }$ at each temperature, is available at Supplementary Data File, https://links.lww.com/IN9/A7.

### 2.3 Metabolite sensitivity analysis

In the absence of direct experimental measurements, we used a biologically justified concentration range of 0.01 to 10 mM. This metabolite concentration is consistent with established systems biology standards for defining physiological metabolite concentrations and thus provides a realistic framework for assessing temperature-dependent metabolic adaptations in human cells ^[19,33]^. Additionally, due to the limited availability of metabolite concentration data, we were only able to determine concentration ranges for selected metabolites across different immune cell types. To evaluate whether these available metabolites significantly influence the MDF and ΔrG values, we performed a sensitivity analysis using the concept of shadow price ^[34]^.

The shadow price, derived from optimization theory, represents the rate at which the optimal value of an objective function (in this case, the MDF) changes in response to a small perturbation in a constraint (eg, a metabolite concentration bound and/or reaction bounds). In the context of thermodynamic analysis, a high shadow price indicates that a particular metabolite or reaction imposes a strong limitation on the pathway’s thermodynamic feasibility—meaning that small changes in its concentration can substantially affect the MDF value. This approach thus helps identify “bottleneck” metabolites or reactions that exert the greatest control over pathway thermodynamics.

Following the framework described by Noor et al ^[19]^, we computed shadow prices for all metabolites and reactions from each pathway and compiled a list of those to which the MDF was most sensitive. We then compared this list with the metabolites for which experimental concentration ranges were available. Encouragingly, we found that the experimentally characterized metabolites corresponded to those with the highest shadow prices, confirming that our available data captured key thermodynamically influential metabolites. Detailed results of the Shadow Price Analysis are provided in the Supplementary Tables S5 and S6, https://links.lww.com/IN9/A7.

### 2.4 Data integration for different cell types

The metabolomics datasets for macrophages, neutrophils, dendritic cells, and erythrocytes were obtained from Hooftman et al ^[26]^ and Kaiser et al ^[27]^, respectively. For macrophages, absolute metabolite concentrations were directly taken from Hooftman et al ^[26]^. In contrast, for neutrophils, dendritic cells, and erythrocytes, Kaiser et al ^[27]^ reported metabolite levels as log fold changes (logFC). Accordingly, we applied the same logFC ratios to adjust the concentration ranges derived from reference values. A complete list of metabolites and their corresponding concentration ranges is provided in the Supplementary Tables S2 and S3, https://links.lww.com/IN9/A7.

### 2.5 Ethics statement

This is a computational work and metabolomics datasets are from previously published papers ^[26,27]^. No ethics committee approval was required for this study.

## 3. Results

### 3.1 Overall driving force of pathways remain nearly constant

MDF analysis is a powerful tool for identifying the bottleneck and critical reactions in metabolic pathways, especially under conditions where temperature fluctuations may impact thermodynamic feasibility ^[19,32]^. While human metabolism can adjust to minor temperature changes through homeostatic mechanisms, significant or prolonged deviations highly disrupt metabolic efficiency, enzyme function, and energy balance, ultimately compromising health ^[29]^. Hence, examining changes in ∆G offers unique insights into the effects of temperature fluctuations on metabolic feasibility. Since ∆G is temperature-dependent, it directly influences the thermodynamic feasibility of metabolic reactions (as shown in Equation 1) ^[35]^.

To this end, we applied MDF analysis to obtain the overall driving force of pathways and ∆G of each reaction with the substrate concentration range set between 0.01 and 10 mM. Due to the lack of experimental measurements, a biologically justified concentration range was used as it represents the metabolite concentration levels across diverse human states, from nutrient-rich to starved conditions ^[19,36]^. This range is widely accepted in metabolic modeling and experimental studies, allowing flexibility to capture low- and high-concentration metabolites relevant to various pathways ^[5,19,21]^. Additionally, it aligns with established standards in systems biology for representing physiological concentrations and thus allows for a realistic assessment of how human cells adapt to temperature fluctuations ^[19]^. We also increased the temperature from 310.15 to 314.15 K to obtain the overall driving force of the pathways and ∆G changes of each reaction.

**Figure 1.**
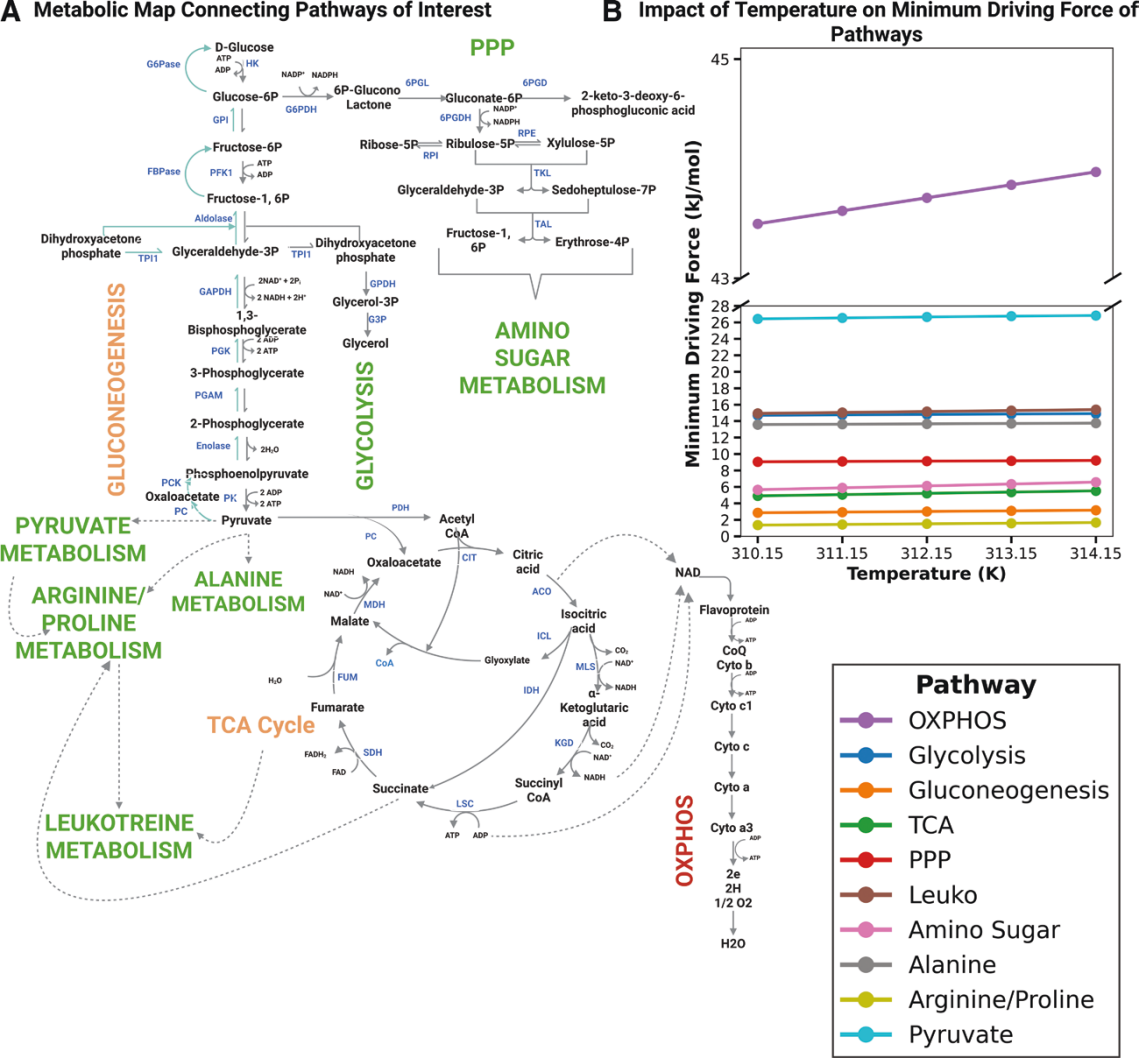
(A) Pathway map with the pathways with the highest driving force highlighted in red, medium driving force highlighted in orange, and the lowest driving force highlighted in green. (B) The minimum driving force of the pathways when temperature increases from 310.15 to 314.15 K for the physiological substrate concentration ranging from 0.01 to 10 mM remains almost steady, showing that the impact of the temperature shift is mainly found in metabolite/reaction level. This figure was created using Biorender (https://BioRender.com/i41q165). OXPHOS, oxidative phosphorylation; PPP, pentose phosphate; TCA, tricarboxylic acid.

We calculated the minimum driving force of each pathway, which is the smallest free energy change needed for a pathway to be thermodynamically feasible. Our initial question was whether temperature changes have a drastic impact on the MDF of metabolic pathways, whether a pathway could shift dramatically from being highly feasible to potentially infeasible (or vice versa) as temperature varies. As expected, all pathways exhibited relatively consistent driving forces across the tested temperature range (310.15–314.15 K). This observation does not imply that temperature has no influence on cellular behavior. Rather, as fever elevates metabolic demand, cells likely accelerate their fluxes to meet this increased demand ^[19]^. This adjustment leads to a re-equilibration of the metabolite pools, effectively compensating for temperature-induced perturbations and maintaining an approximately constant MDF. This trend is evident in Figure 1b, which shows nearly constant MDF of all pathways as the temperature changes. These findings led us to examine which metabolic pathways experienced the strongest temperature-induced effects. Hence, we expected to observe significant downstream shifts to maintain the steady MDF for all pathways. However, comparing the minimum driving forces of different pathways, we found pathways such as arginine/proline metabolism to have the lowest driving force, pathways such as gluconeogenesis and TCA showed moderate MDF, and the rest of the pathways showed more than 5 kJ/mol MDF, which is considered high. However, the highest minimum force required as per our analysis was found to be OXPHOS, with pyruvate metabolism having second highest value.

### 3.2 Pathways whisper but reactions scream as temperature changes

**Figure 2.**
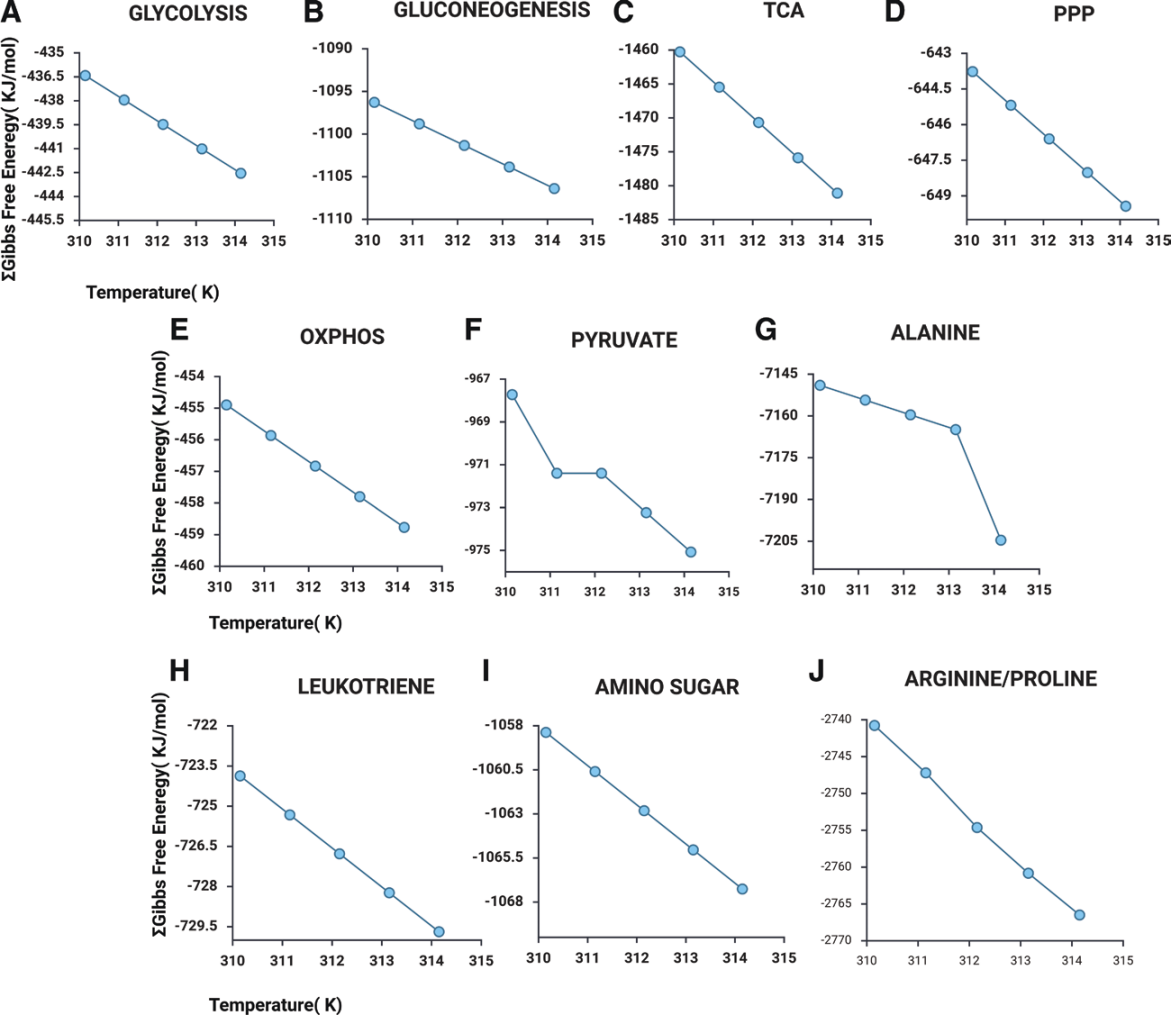
Sum of Gibbs free energy change of all reactions in all pathways shows that despite a minimum driving force remaining almost steady, the Gibbs free changes. (A–J) The overall thermodynamic feasibility of each pathway shows significant changes, highlighting a steep change originating from arginine/proline metabolism. This figure was created using Biorender https://BioRender.com/i41q165. OXPHOS, oxidative phosphorylation; PPP, pentose phosphate; TCA, tricarboxylic acid

The nearly constant MDF of the pathways came at a tremendous cost at the reaction level. The MDF enables identification of thermodynamic bottlenecks, that is, the least feasible reactions under varying temperatures, while the sum of all ∆G values provides a measure of overall pathway spontaneity, allowing comparison of thermodynamic favorability across pathways. We observed the highest feasibility from arginine and proline metabolism, which acts as a fuel supply at the time of increased metabolic demand due to temperature increase, supplemented by still high level of activity in energy metabolism such as pyruvate, TCA, OXPHOS, PPP, and amino-sugar metabolism and finally lowest activity from gluconeogenesis, suggesting fever thermodynamically reprograms the metabolism (Figure 2). With the temperature increase, the highest feasibility from amino acid-related pathways signifies metabolic reprogramming to enhance catabolism, to meet the increased energy demand. In addition, distinct amino acids are known to play specific roles in host response. We next posed the following questions. What is occurring at the reaction level? Which specific reactions do bear the brunt of emerging metabolic demand?

Although a specific pathway may exhibit an overall positive driving force and negative net ∆G, the individual reactions within it may behave differently from one another. Some reactions can be highly thermodynamically favorable at a certain temperature, and that might change with temperature fluctuations. Our MDF predictions are in accordance with the reported thermodynamic nature of pathways. Additionally, the reactions that showed the highest deviation in ∆G originated mainly from glycolysis, gluconeogenesis, and arginine and proline metabolism. Glycolytic reactions such as GPI (glucose-6 phosphate to fructose 6 phosphate), HK (formation of glucose-6-phosphate), and PK (phosphoenolpyruvate to pyruvate) were found to have high net −∆G values from the initial temperature starting at 310.15 K and maintained high values till 314.15 K. The observation that pyruvate production is the most thermodynamically feasible reaction in the glycolytic pathway aligns with findings from several studies ^[5,20]^, reinforcing the reliability of using MDF analysis for further predictions and exploration. Additionally, the high feasibility of initial steps of glycolysis, mainly the conversion of glucose to the next intermediates, suggests the increased glycolytic dependency with the onset of fever, as reported by several studies, such as Troha et al ^[37]^, and O’Sullivan et al ^[38]^.

Significant metabolic shifts were observed within the arginine and proline metabolic pathway, particularly in reactions such as carbonate hydro-lyase (carbon-dioxide-forming), l-arginine carboxy-lyase, and l-proline:2-oxoglutarate. Interestingly, each of these reactions involves CO_2_ generation, and the calculated ∆G values consistently shifted to favor CO_2_ production. This thermodynamic trend is consistent with an increase in metabolic activity, wherein enhanced flux through CO_2_-yielding reactions reflects elevated oxidative and catabolic processes. Additionally, the reaction catalyzed by 4-hydroxy-2-oxoglutarate glyoxylate-lyase (pyruvate-forming) exhibited a pronounced decrease in thermodynamic feasibility with increasing temperature. Interestingly, d-aspartate:2-oxoglutarate aminotransferase, which produces 4-hydroxy-2-oxoglutarate and aspartate, became increasingly favorable under the same conditions. As all these reactions are localized within the mitochondria, these observations suggest a temperature-dependent modulation of mitochondrial metabolic capacity. This interpretation aligns with the findings of Heintzman et al ^[18]^, who reported temperature-induced alterations in mitochondrial function in T cells ^[18]^.

By integrating the overall thermodynamic driving force and assessing the maintenance of a steady MDF, we identified temperature-dependent reorganization of mitochondrial metabolism, a phenomenon that has been largely reported by recent studies ^[1,18,38]^. These findings raise a critical question: do distinct cell types exhibit conserved or divergent thermometabolic responses? Furthermore, if such divergence exists, is there a central regulatory mechanism coordinating these temperature-mediated shifts in immune cell metabolism?

### 3.3 Cell-type-specific thermodynamic responses

We further wanted to explore the change in thermodynamic feasibility of reactions in different immune cells, such as macrophages, neutrophils, erythrocytes, and dendritic cells. These immune cells are often the first responders against any disease condition and show highly versatile metabolic behavior. Based on the study by Hooftman et al ^[26]^, various metabolite concentrations were measured in response to lipopolysaccharide (LPS) induction in macrophages. Using this data, we adjusted the concentration constraints for metabolites like ATP, Adeniosine Diphosphate, Uridine Diphosphate, fumarate, itaconate, and succinate (the complete list of metabolite constraints can be found in Supplementary Tables S2 and S3, https://links.lww.com/IN9/A7). We then observed how these adjustments affected the changes in −∆G across the nine pathways discussed earlier. LPS induction is the process of exposing macrophages to LPSs, which are large molecules found in the outer membrane of gram-negative bacteria ^[17]^. This process acts as a potent activator in macrophages and activates immune responses against bacterial infection ^[17]^. Hence, analyzing the change in feasibility under these conditions allows us to decipher the proper immune response of macrophages against bacterial infection. Similarly, the metabolite concentration trends reported by Kaiser et al ^[27]^, for neutrophils, erythrocytes, and dendritic cells—specifically metabolites like ornithine, putrescine, and arginine (the complete list is provided in the Supplementary Tables S2 and S3, https://links.lww.com/IN9/A7)—enabled us to perform MDF analysis on four pathways: arginine/proline, alanine, amino sugar, and leukotriene metabolism.

Based on our analysis, the overall category (ie, high, medium, and low) to which each pathway belongs remained the same, with a small increase or decrease in minimum driving forces, while individual reactions showed unique responses as noted earlier. Specifically, reactions involving glutamate utilization seemed to be the most sensitive across different immune cell types to the temperature increase. To determine what a significant change in ∆G is, we followed the same criteria as described by Noor et al, ^[19]^ where a change that exceeds approximately 3 kJ/mol indicates a meaningful shift in reaction favorability ^[7]^. Supplementary Table S4, https://links.lww.com/IN9/A7, shows the reactions with significant changes in the ∆G values in macrophages, neutrophils, erythrocytes, and dendritic cells in comparison to the normal state described earlier. Some of the reactions mentioned earlier, namely GPI and HK (the formation of fructose-1,6-biphosphate and glucose-6-phosphate), are key metabolites needed for the glycolytic switch during the immune response of macrophages ^[20]^. Similarly, numerous metabolites such as pyruvate, various glutamate compounds, and ornithine are important precursors for various amino acid metabolisms involved in neutrophil, dendritic cells, and erythrocytes ^[29]^ metabolism. The change in feasibility of the reaction (shown in Supplementary Table S4, https://links.lww.com/IN9/A7) involved in the conversion of arginine to ornithine and urea, conversion of arginine to citrulline, and the formation of agmatine, was found to be more thermodynamically feasible for dendritic and erythrocytes in comparison to neutrophils. The ∆G values observed across different cell types indicate that the urea cycle plays diverse and cell-specific roles, as widely reported in literature ^[36,39]^. Specifically, this includes the conversion of arginine into ornithine and urea or into citrulline and ammonia. Hence, by analyzing shifts in ∆G, we can gain valuable and reliable insights into the unique metabolic capabilities of each cell type ^[40]^.

**Figure 3.**
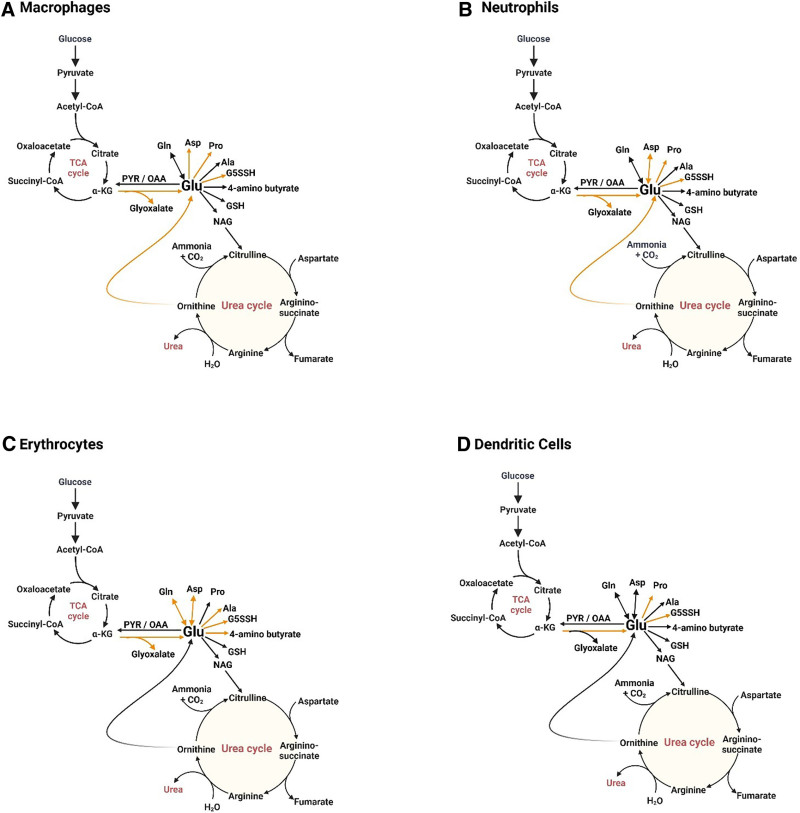
Highlighted reactions that show the highest variation in Gibbs free energy with temperature increase. Orange color denotes the reactions whose Gibbs free energy varies by more than 3 kJ/mol with a change in temperature. All the identified reactions for different cell types can be linked to glutamate metabolism. (A) Metabolic map of reactions associated with glutamate metabolism that show a change in Gibbs free energy with temperature increases in LPS-induced macrophages. Reactions identified lead to the production of other amino acids, such as proline, aspartate, and important compounds like G5SSH. (B) Metabolic reactions that are most sensitive to temperature increase in neutrophils, which include amino acids such as aspartate, alanine, and proline, and also the production of glutamate through ornithine. (C) Erythrocyte’s metabolic reactions that show significant Gibbs free energy shift consist of glutamate and glutamine, aspartate, alanine, proline, and 4-aminobutyrate. (D) Dendritic cells metabolic shift, as predicted by Gibbs free energy, was found to be limited to mainly proline and G5SSH-associated reactions. This figure was created using Biorender (https://BioRender.com/i41q165).

Next, we analyzed the reactions with the highest change in ∆G changes with temperature increase within each cell type and found that the reactions involving glutamate show distinct increases/decreases. Glutamate metabolism is known to be essential for cellular and physiological functions because of its key roles in various amino acid metabolism. Glutamate is a major neurotransmitter in the brain and a precursor for several critical biomolecules ^[41,42]^. We identified the change in the feasibility of reactions involved with glutamate metabolism (directly or indirectly) as a driving force for each immune cell to acquire its unique function. Figure 3 highlights the reactions in each cell type that show a significant change in their ∆G value with the temperature fluctuations. We found the fate of glutamate compounds to be an important factor in determining the function of the cell. For example, in macrophages, the reaction, p5c dehydrogenase feasibility increases by more than 6 kJ/mol after 2 degrees rise in temperature but in the direction where glutamate is used to produce G5SSH whereas the reaction, ornithine aminotransferase (OAT), where ornithine and alpha-ketoglutarate produce glutamate and G5SSH show more than 6 kJ/mol decrease in ∆G value after 2-degree rise in temperature. The same reactions, P5C dehydrogenase and OAT, exhibit similar behavior in neutrophils and erythrocytes. However, in dendritic cells, the feasibility of P5C dehydrogenase decreases with an initial rise in temperature and continues to remain below its starting value with each subsequent degree increase. In contrast, the feasibility of the OAT reaction increases with the initial temperature rise and continues to grow steadily up to 314.15 K. Similar analysis reveals the ∆G value for the reactions involved in proline production increases for all temperatures except for the first temperature increase (311.15 K). Overall, how a cell utilizes glutamate to form different products such as ornithine, aspartate, alanine, and 4-aminobutyrate (also known as GABA) plays a crucial role in determining the function of a cell type. For example, the change in thermodynamic feasibility of the reaction involved with ornithine in macrophages and neutrophils supports the antimicrobial activity delineated by both cells. Whereas erythrocytes and dendritic cells show more than 6 kJ/mol increase in ∆G value for the production of glyoxylate and pyruvate, along with a decrease in ∆G^o^ value of the reaction-producing aspartate. Hence, we postulate that the fate of glutamate and reprogramming in glutamate metabolism are significant drivers of regulating proper immune responses of different cell types, namely macrophages, neutrophils, dendritic cells, and erythrocytes.

## 4. Discussion

A thermodynamic approach, such as MDF analysis, offers valuable insights into how metabolic and enzymatic pathways in immune cells adapt to thermal fluctuations, including conditions like fever. In this study, we applied MDF analysis to investigate the impact of temperature increases on nine distinct pathways and examined the thermodynamic feasibility of these pathways across different immune cell types. This study, to the best of our knowledge, is the first to report temperature-dependent changes in the thermodynamic feasibility of individual reactions, as well as to assess overall pathway feasibility within a human metabolic system. We further sought to determine whether pathway-level feasibility necessarily reflects consistent trends across all constituent reactions. By integrating reaction-level and pathway-level analyses, this work provides deeper insight into the metabolic adjustments underlying the fever response and how different immune cell types may differentially adapt to elevated temperatures.

Temperature plays a key role in the thermodynamics of metabolic reactions that are critical for maintaining physiological balance, optimizing metabolic engineering processes, and informing clinical practices ^[1]^. ∆G is fundamental for assessing reaction feasibility in various biological and biotechnological contexts as it quantifies the spontaneity of a reaction ^[4]^. Hence, by using MDF analysis, we can quantify the spontaneity of reactions at different temperatures and metabolite concentrations. To conduct MDF analysis, accurate estimates of ∆G^o^ for all reactions in the pathway of interest are required ^[19]^. Among the various databases and tools available, such as National Institure of Standards and Technology ^[43]^, Metabolic Encyclopedia, Kyoto Encyclopedia of Genes and Genomes, and Thermodynamic Metabolic Modeling, Equilibrator stands out due to its unique combination of features, accessibility, and versatility ^[44–47]^. Equilibrator leverages a curated and validated thermodynamic database derived from multiple sources, ensuring high reliability for biochemical reactions ^[19]^. It also implements the component contribution method, which combines experimental data with predictive models, enabling accurate ∆G^o^ estimates even for reactions with sparse data ^[4]^. Additionally, the Equilibrator is specifically designed for biochemical systems, unlike other tools that often focus on general chemistry or reaction mechanisms ^[48]^. However, despite its extensive database, Equilibrator’s reliance on available experimental data limits its application only to specific pathways ^[30]^

To this end, we were able to analyze eight full pathways and one compilation of amino acid/fatty acid reactions for which ∆G^o^ values could be estimated through equilibrator ^[14]^. The pathways in this study (such as glycolysis, TCA, OXPHOS, amino acid metabolisms, etc.) are crucial for energy metabolism and immune response regulation. While a greater number of pathways and reactions would provide valuable insights, the nine pathways we have included in this study delineate key metabolic capabilities and immune responses at different temperatures. We started our analysis with a normal body temperature of 310.15 K and went up to 314.15 K, which is known to be deleterious to health.

One of the challenges in conducting such a thermodynamic analysis is to be able to gather proper concentration data. In this study, we used a range of 0.01 to 10 mM for normal conditions, and to be able to capture the immune cell phenotypes, we used the reported metabolite concentration trends reported by Hooftman et al ^[26]^ for LPS-induced macrophages and Kaiser et al ^[27]^ for neutrophil, dendritic cells, and erythrocytes. While we could not use specific concentrations for all the metabolites involved in the nine pathways mentioned above, the list of metabolites we obtained from these two studies are highly significant metabolites such as ATP, Adeniosine Diphosphate, and various amino acids such as arginine, glutamate, and proline that play crucial roles in metabolism and immune response and were also identified as bottlenecks by our shadow price analysis. Changing the concentration of these metabolites showed a significant impact on the feasibility of the pathways and the associated reactions, which allowed us to garner insights into how the metabolic shift occurs with the temperature increase. However, further advancements in tools for standard Gibbs free energy prediction and metabolite concentrations for different cell types will enable more comprehensive thermodynamic analysis. This will enable the proper study of complex immune responses, which could potentially lead to the identification of effective and novel therapeutic targets.

Here, we highlight that driving force remains steady, net thermodynamic feasibility of pathways changes, and the feasibility of each reaction is where we see most dynamic shifts. We found that the overall feasibility of all pathways increased with temperature; however, reaction feasibility differed, highlighting reactions from glycolysis, gluconeogenesis, arginine/proline, pyruvate, and PPP that could be significant for normal physiological activity and for immune responses. In doing so, we identified glutamate compound derivatives, namely glutamate-5-semialdehyde and ornithine, to show a significant change in thermodynamic feasibility across the mentioned cell types. Glutamate-5-semialdehyde is an important intermediate in the biosynthesis of proline, which ultimately contributes to the formation of GABA. In human immune cells, the conversion of glutamate to glutamate-5-semialdehyde, and subsequently to ornithine and other amino acids, plays a significant role in regulating metabolic pathways that support immune responses. These pathways are especially relevant under conditions of cellular stress, where metabolites such as proline and GABA may help modulate oxidative stress and support immune cell function.

Several studies, such as Ginguay et al, ^[40,41]^ investigated the role of glutamate and its derivatives in modulating the human immune response and regulating fate determination. However, the specific utilization of glutamate to form different compounds within immune cells has not been as exhaustively explored ^[49]^. Glutamate, derived from glutamine through glutaminolysis, plays roles in amino acid synthesis and as a neurotransmitter. In immune cells, glutamate can be further metabolized into compounds such as α-ketoglutarate, which enters the TCA cycle, influencing energy production and biosynthesis ^[50]^. Additionally, glutamate serves as a precursor for the synthesis of glutathione, a vital antioxidant that protects immune cells from oxidative stress ^[51]^. Emerging research highlights the importance of glutamate metabolism in immune function. For instance, the conversion of glutamate-derived metabolites like fumarate has been linked to epigenetic reprogramming in monocytes, enhancing their pathogen responsiveness, a process termed “trained immunity” ^[51]^. While glutamine metabolism is well-characterized in immune responses, the specific roles of glutamate in supporting cellular functions in macrophages, neutrophils, dendritic cells, and erythrocytes are only beginning to be understood. Through thermodynamic analysis of reactions and pathways, we emphasize the potential significance of glutamate metabolism in immune cell functionality. Supplementary Table S4, https://links.lww.com/IN9/A7 provides a detailed overview of reactions showing distinct shifts in Gibbs free energy, underlining the energetic and regulatory importance of glutamate in modulating immune responses.

Overall, this study leverages MDF analysis to provide robust insights into pathway thermodynamics and reaction feasibility, accurately capturing temperature-induced shifts. We categorized the pathways into three distinct groups and identified key reactions that exhibit dynamic changes with increasing temperature. Our findings also suggest that glutamate metabolism plays a pivotal role in modulating immune responses across different immune cell types. Through this work, we demonstrate the value of thermodynamic analyses such as MDF in exploring the complexity of human metabolism and guiding the activation of appropriate immune responses. In the future, this approach can be expanded by integrating additional metabolomics data across diverse cell types and incorporating whole-network metabolic models for deeper system-level insights.

## Author czontributions

SM and RS worked on concept development for this work and developed methodologies. The analysis, followed by the writing and editing, was done by SM and RS.

## Conflicts of interest

The authors declare that they have no conflicts of interest.

## Funding

The funding support is from the National Institute of Health (NIH) R35 MIRA grant (5R35GM143009), awarded to RS.

## Acknowledgments

We gratefully acknowledge the Holland Computing Center (HCC) of the University of Nebraska, which receives support from the Nebraska Research Initiative (United States of America).

## Supplementary Material


