# Supplementary material for: Pathway thermodynamic analysis postulates change in glutamate metabolism as a key factor in modulating immune responses
Source: Immunometabolism (Cobham). 2026 Feb 25;8(1):e00077. doi: 10.1097/IN9.0000000000000077 (PMC12940620; doi:10.1097/IN9.0000000000000077)
Supplement: Supplementary file 1 [file in9-8-e00077-s001.pdf]

Supplementary File for the study “Pathway Thermodynamic Analysis Identifies Change in Glutamate Metabolism as a Key Factor in Modulating Immune Responses”

Table 1: Standard gibbs free energy for the reactions of the nine pathways analyzed in the study. The $\Delta_f G^o$ are obtained from the equilibrator tool and the reactions are from Human1 model (Robinson et al., 2020).

Glycolysis/Gluconeogenesis	$\Delta_f G^o$	TCA	$\Delta_f G^o$	Pyruvate	$\Delta_f G^o$	PPP	$\Delta_f G^o$	OXPHOS	$\Delta_f G^o$	Leukotriene	$\Delta_f G^o$	AminoSugar	$\Delta_f G^o$	Other Amino Acid	$\Delta_f G^o$	Alanine/Proline
'MAR03905'	18.1	'MAR00710'	-11.7	'MAR04193'	24.8	'MAR04052'	3.2	'MAR03975'	-33.6	'MAR01080'	25.8	'MAR03988'	-29.6	'MAR00156'	-3.8	'MAR03807'
'MAR03907'	18.1	'MAR03787'	12.6	'MAR04280'	23.7	'MAR04304'	-6.8	'MAR03977'	-33.6	'MAR01081'	17.4	'MAR04124'	-24.1	'MAR00217'	-13.5	'MAR03819'
'MAR04097'	-8.2	'MAR03957'	-11.7	'MAR03855'	23.4	'MAR04306'	-6.8	'MAR03979'	-33.6	'MAR01085'	-17.8	'MAR04159'	1.5	'MAR00409'	-12.8	'MAR03820'
'MAR04099'	-8.2	'MAR03958'	-11.7	'MAR08512'	20.6	'MAR04350'	-27	'MAR06911'	-33.6	'MAR01100'	15.6	'MAR04299'	-15.8	'MAR02949'	-13.5	'MAR04786'
'MAR04137'	-34.2	'MAR04111'	-27.4	'MAR03854'	14	'MAR04351'	2.1	'MAR06912'	-33.6	'MAR01101'	15.6	'MAR04300'	-15.6	'MAR02996'	-12.8	'MAR11399'
'MAR04281'	23.7	'MAR04112'	-27.4	'MAR08502'	7.6	'MAR04352'	2.1	'MAR06916'	-46.8	'MAR01102'	15.6	'MAR04494'	-11.3	'MAR04156'	-9.1	'MAR04191'
'MAR04388'	23.7	'MAR04113'	-27.4	'MAR03853'	6.3	'MAR04354'	-11.5	'MAR06921'	82.3	'MAR01103'	15.6	'MAR04524'	-12	'MAR04295'	-9.1	'MAR08425'

													.1			
'MAR04283'	-55.6	'MAR04139'	26.5	'MAR04101'	-3.8	'MAR04398'	3.2			'MAR01105'	15.6	'MAR04525'	-40.3	'MAR02190'	3.3	'MAR08416'
'MAR08357'	-55.6	'MAR04141'	26.5	'MAR04103'	-3.8	'MAR04404'	10.3			'MAR01106'	15.6	'MAR04526'	-40.3	'MAR02191'	-15.6	'MAR08603'
'MAR04379'	-17.8	'MAR04145'	38.8	'MAR04087'	-5	'MAR04473'	-6.8			'MAR01287'	-372.5	'MAR04527'	3.7	'MAR02193'	-0.4	'MAR03809'
'MAR04301'	-17.4	'MAR04147'	1.5	'MAR04089'	-5	'MAR04474'	-6.8			'MAR04796'	-19.1	'MAR04528'	-13.7	'MAR02194'	-56.2	'MAR06955'
'MAR04355'	3.9	'MAR04149'	-8	'MAR04091'	-5	'MAR04476'	-12.5					'MAR04529'	65.6	'MAR00709'	-46.8	'MAR04073'
'MAR04358'	25	'MAR04152'	-1.2	'MAR04143'	-7.2	'MAR04477'	-3.4					'MAR04530'	-29.9	'MAR00457'	-15.4	'MAR04785'
'MAR04360'	-36.8	'MAR04209'	-24.8	'MAR08497'	-16	'MAR04501'	3.8					'MAR04628'	-26.2	'MAR00460'	-15.4	'MAR04212'
'MAR04363'	-3.8	'MAR04408'	3.4	'MAR08504'	-43.5	'MAR04565'	-0.9					'MAR04629'	-25.8	'MAR03839'	30.8	'MAR04422'
'MAR04365'	-4.5	'MAR04410'	3.4	'MAR03857'	-51.1	'MAR04567'	-16.6					'MAR07696'	1.8	'MAR03841'	10.9	'MAR08605'

'MAR04368'	19.5	'MAR04454'	6.8	'MAR08511'	-51.1	'MAR09799'	-19					'MAR07697'	-47.5	'MAR03843'	-27.5	'MAR03806'
'MAR04370'	-66.3	'MAR04456'	6.8	'MAR04093'	-52.1	'MAR09800'	-14.6					'MAR07698'	2.2	'MAR03845'	6.7	'MAR03816'
'MAR04371'	-39.4	'MAR04458'	8.5	'MAR04095'	-52.1	'MAR04623'	-25.4					'MAR08373'	-9.4	'MAR03847'	32.8	'MAR08426'
'MAR04373'	18.3	'MAR04465'	28.2	'MAR01568'	-55.6	'MAR04625'	-25.4					'MAR08375'	-29.6	'MAR03849'	-87.6	'MAR04605'
'MAR04375'	6	'MAR04585'	15.7	'MAR08516'	-76.7	'MAR04710'	-26.7					'MAR08376'	-29.6	'MAR03852'	-111.3	'MAR03832'
'MAR04377'	-28.9	'MAR04586'	15.7	'MAR08517'	-76.7	'MAR08074'	25.6					'MAR08672'	-24.1	'MAR03856'	14	'MAR01523'
'MAR04381'	2.6	'MAR04587'	15.7			'MAR08653'	-6.8					'MAR08674'	-15.4	'MAR03860'	32.8	'MAR04424'
'MAR04391'	-5.6	'MAR04589'	1.6									'MAR08675'	-4.1	'MAR03883'	-47.3	'MAR08608'
MAR04394'	-20.4	'MAR05294'	-13.3											'MAR03974'	-41.2	'MAR00442'
'MAR04396'	-7.4	'MAR05297'	-28.2											'MAR04198'	4.2	'MAR06958'

'MAR04521'	-26.4	'MAR07704'	-162											'MAR04200'	-6.1	'MAR06959'
'MAR07747'	-15.6	'MAR07706'	-88.3											'MAR04284'	-7.1	'MAR06961'
		'MAR08743'	42.6											'MAR04348'	-41.2	'MAR06962'
		'MAR08773'	-4.3											'MAR04466'	1.6	'MAR08604'
		'MAR08774'	-34.7											'MAR04467'	4.2	'MAR04423'
		'MAR08775'	-66.8											'MAR04582'	-0.4	'MAR04776'
		'MAR08777'	-34.7											'MAR04696'	14.6	'MAR04777'
		'MAR08778'	-66.8											'MAR04697'	-42.4	'MAR01649'
		'MAR08779'	41.1											'MAR04698'	-42.4	'MAR03993'
		'MAR08781'	-10.9											'MAR04742'	-0.4	'MAR02023'
		'MAR08782'	28.2											'MAR04788'	-8.4	
														'MAR04	-10	

														789'	4.5	
														'MAR04791'	-8.4	
														'MAR04792'	6.7	
														'MAR06409'	1.6	
														'MAR07702'	41.1	
														'MAR07703'	-88.3	
														'MAR08440'	-42.4	
														'MAR09718'	-39.4	
														'MAR09486'	-46.5	
														'MAR03802'	16.3	
														'MAR03804'	16.3	
														'MAR03811'	-4.3	

														'MAR03813'	-5.5	
														'MAR03822'	-43.5	
														'MAR03827'	2.8	
														'MAR03829'	2.8	
														'MAR03831'	-3.9	
														'MAR03862'	-34.6	
														'MAR03865'	-9.3	
														'MAR03870'	29.7	
														'MAR03873'	-8.7	
														'MAR03890'	-15.3	
														'MAR03892'	-31.5	
														'MAR09	-3	

														802'	1.5	
														'MAR03899'	0.2	
														'MAR03903'	-57.1	
														'MAR04109'	0.2	
														'MAR04114'	-20.4	
														'MAR04115'	-20.4	
														'MAR04118'	29.7	
														'MAR04172'	-34.6	
														'MAR04196'	-11.2	
														'MAR04197'	-11.2	
														'MAR04287'	-42.4	
														'MAR04690'	-37.8	

														'MAR04693'	6.5	
														'MAR06780'	0.4	
														'MAR07641'	-115.4	
														'MAR07642'	0	
														'MAR08626'	-23.2	
														'MAR08628'	-28.8	
														'MAR04285'	-42.4	

Table 2: List of metabolites and their concentrations obtained from Hooftman et al, 2023[29] for macrophage.

Metabolites	Units as describe by the authors of Hooftman et al., nature, 2023
2/3-phosphoglycerate	3134885
2-aminoadipate	3590073
2-hydroxyglutarate	52623485
2-ketoglutarate	65912712
3-phosphoserine	413007.7
4-guanidinobutanoate	52698503
5-aminoimidazole ribotide	0
acetylcholine	3.94E+08
acetyl-CoA	713107.7

aconitate	94736361
adenine	10941024
adenosine	2444993
adenylosuccinate	0
ADP	33353890
ADP-hexose	453114
ADP-ribose	718845.9
AICAR	584371.9
alanine	4.32E+08
aminobutyrate	98353423
AMP	15405844
arginine	6.07E+08
argininosuccinate	187971.7
asparagine	6408281
aspartate	6.14E+08
ATP	17452619
beta-alanine	3.92E+08
betaine	7.04E+09
carboxyglutamate	1208876
carnitinamide	56035147
carnitine	1.55E+09
carnosine	1321606
CDP	0
CDP-ethanolamine	4949464
choline	2648602
citicoline	0
citrate	17932083
citrulline	1702383
CMP	0
CMP-2-aminoethylphosphonate	2256831
CMP-acetylneuraminic acid	6704854
creatine	27511.95
creatinine	1.39E+09
cyclic ADP-ribose	3.42E+08
cystathionine	34647954
cysteic acid	6237265
cystine	45152053
cytidine	18469624
cytosine	3370142
dCTP	21581232
dihydroorotate	1024180

dihydroxyacetone phosphate	1850316
dimethylarginine	1.42E+09
dimethyllysine	325740.1
FAD	3.14E+08
formiminoglutamate	14954448
fumarate	1300592
GDP	3408980
GDP-hexose	19151398
glucuronate	1.86E+10
glutamate	5.84E+08
glutamine	6733934
glutamylcysteine	136278.7
glutamylglutamate	241634.4
glutamylglycine	10839736
glutamylproline	3763146
glutathione oxidized	6732261
glutathione reduced	104459.6
glyceraldehyde 3-phosphate	1815420
glycerol 3-phosphate	1349681
glycerophosphoethanolamine	32840516
glycerophosphoinositol	2408984
glycerophosphorylcholine	563209.4
glycerophosphoserine	3757404
glycinamide ribonucleotide	21171079
glycinamide ribonucleotide (GAR)	1484703
glycine	134549.5
glycylglycine	3131151
GMP	1.47E+09
guanidinoacetate	2.69E+09
guanosine	4996912
hippurate	80478.12
histidine	3387921
homoarginine	2922146
hydantoin-5-propionic acid	604235.7
hydroxyphenyllactate	1.12E+08
hydroxyproline	1595258
hydroxytrimethyllysine	7707584
hypotaurine	68525139
hypoxanthine	9700572
imidazole propionate	1.91E+09
IMP	1530820

indole	6376308
indole-3-lactate	6253933
inosine	5213966
inositol	446259.6
isoleucine	676012.1
itaconate	67196758
ketoleucine/ketoisoleucine	0
kynurenine	14202210
lactate	8.2E+08
leucine	4924156
lysine	2283195
malate	9608399
malonate	39549953
methionine	1040075
methionine sulfoxide	31269426
methylmalonate	8819044
N-acetylarginine	5706693
N-acetylaspartate	286737.4
N-acetylglutamate	309182.2
N-acetylglutamine	993609.4
N-acetylhexosamine phosphate	64533.78
N-acetylhistidine	89034522
N-acetylneuramic acid	1.09E+09
N-acetylornithine	23258693
N-acetylputrescine	5612131
N-acetylserine	978539
N-acetyltaurine	4.17E+08
NAD	9.27E+08
NADH	1.41E+08
NADP	3.53E+08
NADPH	3889951
N-carbamoylaspartate	2.7E+08
N-carbamoylglutamate	8509604
N-formylkynurenine	13724862
nicotinamide	991025.9
nicotinamide mononucleotide (NMN)	82152862
nicotinamide riboside	9311290
ophthalmate	4001315
ornithine	1917583
orotate	51106085
orotidine	2.23E+08

pantothenate	15506273
pentose phosphate	1738643
phenylalanine	44597590
phosphocreatine	12759117
phosphoenolpyruvate	46403726
phosphoethanolamine	180687.7
phosphorylcholine	555414.2
proline	1075298
propionylcholine	1393453
pyridoxine	745706.4
pyroglutamic acid	822098.4
pyruvate	1.2E+08
S-(2-succinyl)cysteine	150133.3
S-adenosylhomocysteine	3143711
S-adenosylmethionine	8582305
sedoheptulose 7-phosphate	16260829
serine	2046034
succinate	25011447
succinyladenosine	60178944
taurine	1303801
thiamine	7.36E+08
threonine	1.35E+08
trimethyllysine	2007042
tryptophan	66247009
tyrosine	9.91E+08
UDP	5.26E+08
UDP-GlcNac	35935904
UDP-glucuronic acid	19202262
UDP-hexose	1.33E+09
UMP	21027195
uracil 5-carboxylate	230817.3
uric acid	485563
uridine	24265281
UTP	332205
valine	11276440
xanthine	20082039
xanthosine	0
**Argininosuccinate**	3E+09
**Glutamine**	61642193
**GABA**	2.44E+08
**Asparagine**	20163517

**Glutamate**	82136381
**Aspartate**	3.66E+08
**Succinate**	11204267
**aKG**	35809121
**Malate**	1145220
**Fumarate**	17109305
**Ornithine**	3606597
**Arginine**	2047476
**Carbamoyl_phosphate**	15953843
**Citrulline**	841827.5

Table 3: Metabolite list obtained from Kraiser et al., 2020 for neutrophils, dendritic cells, and erythrocytes and their reported fold Change.

**Metabolite**	**LogFC: erythrocytes**	**LogFC: dendritic cells**	**LogFC: neutrophils**
Arg	-1.15852	-1.34183	-3.8364
Orn	1.032873	0.923579	0.440181
Ser	-2.09396	-1.54726	-2.84148
Histamine	1.733008	2.526671	8.690358
PEA	1.161706	1.084597	0
Putrescine	1.495052	-0.18757	-0.71198
H1	-2.48262	-1.86428	-1.83933
Taurine	-0.64361	1.934459	5.277249
Ala	0.50933	0.578674	-3.36323
Asn	-0.88248	-0.86303	-3.17453
Asp	0.970047	0.361953	-2.00766
Glu	0.61525	0.360308	-1.90243
Gly	0.480564	-0.32364	-1.54463
His	-0.26592	-0.24553	-3.05664
Ile	-0.55661	-0.44096	-1.89562
Leu	-0.31928	-0.1846	-1.96356
Lys	-0.34991	-0.17975	-2.82296
Met	0.166447	0.232841	-1.97775
Phe	-0.66201	-0.54511	-1.71883
Pro	-0.22541	-0.68951	-2.69163
Thr	-0.85417	-0.6872	-2.00158
Tyr	-0.64362	-0.55487	-1.79594
Val	-0.54943	-0.18861	-1.78543

Table 4: List of reactions originating from different pathways that show a distinct change in thermodynamic feasibility when the concentration of metabolites is changed from normal physiological condition to that obtained from Hooftman et al, 2023[29], and Kaiser et al., 2020[23]. The first column shows the reaction equation including the participating reactants and products, and the second column shows the name of the pathway the reaction originates from. In the third column, the observed change is mentioned such as the change of +ΔGº to -ΔGº value or if a significant increase/decrease is observed and the fourth column indicates the cell type where the specified change was observed. In the final column, the possible contribution of each of these changes in immune responses is mentioned with references.

Reaction	Pathway	Change Observed	Outcome	Cell type
fructose-6-phosphate[c] + UTP[c] => fructose-1,6-bisphosphate[c] + H+[c] + UDP[c]	Glycolysis/gluconeogenesis	+ΔGº to -ΔGº	Enables switch to glycolytic metabolism[31]	Macrophage
1,3-bisphospho-D-glycerate[c] + ADP[c] <=> 3-phospho-D-glycerate[c] + ATP[c]	Glycolysis/gluconeogenesis	more than 4X change in ΔGº value	Precursors for amino acids such as serine, cysteine, glycine, and alanine are crucial for essential cellular functions[37,38].	Macrophage
1,3-bisphospho-D-glycerate[c] + H2O[c] => 3-phospho-D-glycerate[c] + H+[c] + Pi[c]	Glycolysis/gluconeogenesis	More than 10KJ/mol change in ΔGº value	Precursors for amino acids such as serine, cysteine, glycine, and alanine are important for essential cellular functions[37,38].	Macrophage
acetoacetate[m] + succinyl-CoA[m] => acetoacetyl-CoA[m] + succinate[m]	TCA	+ΔGº to -ΔGº	Helps in the generation of the mitochondrial proton gradient	Macrophage

			and ATP synthesis[39]	
isocitrate[m] + NAD+[m] => AKG[m] + CO2[m] + NADH[m]	TCA	+ΔG° to -ΔG°	Helps in the generation of the mitochondrial proton gradient and ATP synthesis[39]	Macrophage
glyoxalate[m] + H+[m] + NADPH[m] => glycolate[m] + NADP+[m]	TCA	+ΔG° to -ΔG°	Suppresses harmful effects of peroxide on mitochondrial activity[40].	Macrophage
H+[c] + NADH[c] + OAA[c] <=> malate[c] + NAD+[c]	TCA	More than 10KJ/mol change in ΔG° value	Helps in the generation of the mitochondrial proton gradient and ATP synthesis[39]	Macrophage
CoA[m] + GTP[m] + succinate[m] <=> GDP[m] + Pi[m] + succinyl-CoA[m]	TCA	Decrease in ΔG° value	Helps in the generation of the mitochondrial proton gradient and ATP synthesis[39]	Macrophage
glycolaldehyde[c] + H2O[c] + NAD+[c] => glycolate[c] + 2 H+[c] + NADH[c]	TCA	Decrease in ΔG° value	Suppresses harmful effects of peroxide on mitochondrial activity[40].	Macrophage
glycolaldehyde[m] + H2O[m] + NAD+[m] => glycolate[m] + 2 H+[m] + NADH[m]	TCA	More than 10KJ/mol change in ΔG° value	Suppresses harmful effects of peroxide on mitochondrial activity[40].	Macrophage
H+[c] + lactaldehyde[c] + NADPH[c] => NADP+[c] + propane-1,2-diol[c]	Pyruvate Metabolism	+ΔG° to -ΔG°	Alterations in carbohydrate metabolism[41].	Macrophage

malate[m] + NAD+[m] => CO2[m] + NADH[m] + pyruvate[m]	Pyruvate Metabolism	+ΔGº to -ΔGº	Precursors for amino acids such as serine, cysteine, glycine, and alanine are very important for essential cellular functions[37,38].	Macrophage
H+[m] + NADH[m] + pyruvate[m] <=> L-lactate[m] + NAD+[m]	Pyruvate Metabolism	-ΔGº to +ΔGº	Helps in up-regulation of glycolysis[31].	Macrophage
malate[m] + NADP+[m] => CO2[m] + NADPH[m] + pyruvate[m]	Pyruvate Metabolism	More than 10KJ/mol change in ΔGº value	Precursors for amino acids such as serine, cysteine, glycine, and alanine are very important for essential cellular functions[37,38].	Macrophage
acetyl-CoA[c] + H2O[c] => acetate[c] + CoA[c] + H+[c]	Pyruvate Metabolism	More than 10KJ/mol change in ΔGº value	Promotes anti-tumor activity in immune cells[42].	Macrophage
2-deoxy-D-ribose-5-phosphate[c] <=> acetaldehyde[c] + GAP[c]	PPP	+ΔGº to -ΔGº	Inflammatory mediator in immune cells[43].	Macrophage
ATP[c] + deoxyribose[c] => 2-deoxy-D-ribose-5-phosphate[c] + ADP[c] + H+[c]	PPP	+ΔGº to -ΔGº	Changes in mitochondrial function, possibly provoking of oxidative stress[44].	Macrophage

5 H+[m] + NADH[m] + ubiquinone[m] => NAD+[m] + ubiquinol[m] + 4 H+[i]	OXPHOS	More than 10KJ/mol change in ΔG° value	Promotes mitochondrial function[31].	Macrophage
O2[r] + AKG[r] + proline[r] => CO2[r] + succinate[r] + trans-4-hydroxy-L-proline[r]	Alanine Proline Metabolism	More than 10KJ/mol change in ΔG° value	Precursors for amino acids such as serine, cysteine, glycine, and alanine are very important for essential cellular functions[37,38].	Macrophage
arginine[c] + 2 NADPH[c] + 2 O2[c] => citrulline[c] + 2 H2O[c] + 2 NADP+[c] + NO[c]	Alanine Proline Metabolism	More than 10KJ/mol change in ΔG° value	Regulation of urea cycle for proper immune response[30].	Macrophage
ATP[c] + glucosamine[c] => ADP[c] + glucosamine-6-phosphate[c] + H+[c]	Amino sugar metabolism	More than 10KJ/mol change in ΔG° value	Inhibition of inflammation[45].	Macrophage
ATP[c] + glucosamine[c] => ADP[c] + glucosamine-6-phosphate[c] + H+[c]	Amino sugar metabolism	More than 10KJ/mol change in ΔG° value	Inhibition of inflammation[45].	Macrophage
glutamate[m] + 2 H+[m] + NADH[m] <=> H2O[m] + L-glutamate 5-semialdehyde[m] + NAD+[m]	Arginine and proline metabolism	More than 6 KJ/mol increase	Precursor to other amino acids, involvement in energy metabolism, and	Neutrophils, Dendritic cells,

			immune cell activation[25].	
AKG[m] + ornithine[m] <=> glutamate[m] + L-glutamate 5-semialdehyde[m]	Arginine and proline metabolism	More than 5 KJ/mol increase	The precursor to polyamine synthesis helps in the regulation of NO cycle[30,31].	Neutrophil;
arginine[c] + H2O[c] => citrulline[c] + H+[c] + NH3[c]	Arginine and proline metabolism	More than 6 KJ/mol increase	Helps in the regulation of the NO cycle and promotes the antimicrobial activity[30].	Neutrophil
glutamyl-5-phosphate[m] + H+[m] + NADPH[m] => L-glutamate 5-semialdehyde[m] + NADP+[m] + Pi[m]	Arginine and proline metabolism	More than 6 KJ/mol increase	Precursor to other amino acids, involvement in energy metabolism, and immune cell activation[25].	Neutrophil
arginine[m] + H+[m] => agmatine[m] + CO2[m]	Arginine and proline metabolism	More than 6 KJ/mol increase	The precursor to polyamines helps in immune modulation[31,32].	Neutrophil
AKG[m] + L-erythro-4-hydroxyglutamate[m] => 4-hydroxy-2-oxoglutarate[m] + glutamate[m]	Arginine and proline metabolism	More than 6 KJ/mol decrease	Precursor to other amino acids, involvement in energy metabolism, and immune cell activation[25].	Neutrophil
acetyl-CoA[m] + glutamate[m] => CoA[m] + H+[m] + N-acetyl-L-glutamate[m]	Arginine and proline metabolism	More than 6 KJ/mol decrease	The precursor to other amino acids essential for cellular function[33,46].	Neutrophil
arginine[m] + H2O[m] => ornithine[m] + urea[m]	Arginine and proline metabolism	More than 7	Balance of NO cycle and	Neutrophil

		KJ/mol increase	maintain proper inflammatory response[47].	
O_2[r] + AKG[r] + proline[r] => CO_2[r] + succinate[r] + trans-4-hydroxy-L-proline[r]	Arginine and proline metabolism	More than 5 KJ/mol decrease	Precursor to other amino acids, involvement in energy metabolism, and immune cell activation[25].	Neutrophil
arginine[c] + 2 NADPH[c] + 2 O_2[c] => citrulline[c] + 2 H_2O[c] + 2 $NADP^+$[c] + NO[c]	Arginine and proline metabolism	More than 6 KJ/mol decrease	Balance of NO cycle and maintain proper inflammatory response[48].	Neutrophil
serine[c] => H^+[c] + NH_3[c] + pyruvate[c]	Glycine, serine and threonine metabolism	More than 5 KJ/mol decrease	Maintains cellular function and acts as a precursor for synthesizing proteins, nucleotides, and other biomolecules[31].	Neutrophil
serine[c] => dehydroalanine[c] + H_2O[c]	Glycine, serine and threonine metabolism	More than 4 KJ/mol decrease	Maintains cellular function and acts as a precursor for synthesizing proteins, nucleotides, and other biomolecules[31].	Neutrophil
arginine[c] + glycine[c] <=> guanidinoacetate[c] + ornithine[c]	Glycine, serine and threonine metabolism	More than 5 KJ/mol decrease	The precursor to polyamine synthesis helps in the regulation of NO cycle[47].	Neutrophil

alanine[m] + glyoxalate[m] => glycine[m] + pyruvate[m]	Glycine, serine and threonine metabolism	More than 6 KJ/mol decrease	Essential for amino acid metabolism[31].	Neutrophil
alanine[x] + glyoxalate[x] => glycine[x] + pyruvate[x]	Glycine, serine and threonine metabolism	More than 5 KJ/mol decrease	Essential for amino acid metabolism[21].	Neutrophil
aspartate[c] + ATP[c] + citrulline[c] => AMP[c] + argininosuccinate[c] + H+[c] + PPi[c]	Alanine, aspartate and glutamate metabolism	More than 5 KJ/mol decrease	A crucial step in incorporating nitrogen into the urea cycle[30,48].	Neutrophil
aspartate[c] => fumarate[c] + H+[c] + NH3[c]	Alanine, aspartate and glutamate metabolism	More than 6 KJ/mol decrease	A crucial step for the deamination of amino acids to aid energy metabolism[49].	Neutrophil
ATP[c] + glutamate[c] + NH3[c] => ADP[c] + glutamine[c] + Pi[c]	Alanine, aspartate and glutamate metabolism	More than 4 KJ/mol decrease	Essential for maintaining nitrogen balance and detoxifying ammonia[33,50].	Neutrophil
AKG[c] + alanine[c] <=> glutamate[c] + pyruvate[c]	Alanine, aspartate and glutamate metabolism	More than 5 KJ/mol increase	Essential for maintaining nitrogen balance and detoxifying ammonia[33].	Neutrophil
AKG[m] + alanine[m] <=> glutamate[m] + pyruvate[m]	Alanine, aspartate and glutamate metabolism	More than 8 KJ/mol decrease	Essential for maintaining nitrogen balance and detoxifying ammonia[23].	Neutrophil
2-oxoglutaramate[c] + H2O[c] => AKG[c] + H+[c] + NH3[c]	Alanine, aspartate and glutamate metabolism	More than 5 KJ/mol decrease	Helps to manage proper ammonia levels and NO[47].	Neutrophil
asparagine[c] + H2O[c] => aspartate[c] + H+[c] + NH3[c]	Alanine, aspartate and glutamate metabolism	More than 7 KJ/mol increase	Helps to manage proper ammonia level[s47].	Neutrophil

glutamine[c] + pyruvate[c] => 2-oxoglutaramate[c] + alanine[c]	Alanine, aspartate and glutamate metabolism	-ΔGº to +ΔGº	Essential for maintaining nitrogen balance and detoxifying ammonia[32,33].	Neutrophil
glutamine[m] + pyruvate[m] => 2-oxoglutaramate[m] + alanine[m]	Alanine, aspartate and glutamate metabolism	+ΔGº to _-ΔGº	Essential for maintaining nitrogen balance and detoxifying ammonia[22,23].	Neutrophil
D-alanine[c] <=> alanine[c]	Alanine, aspartate and glutamate metabolism	0 to - ΔGº		Neutrophil
H2O[c] + N-acetyl-L-aspartate[c] => acetate[c] + aspartate[c]	Alanine, aspartate and glutamate metabolism	More than 4 KJ/mol increase	Helps to manage proper acetate supply[42].	Neutrophil
1-pyrroline-5-carboxylate[c] + H+[c] + H2O[c] <=> L-glutamate 5-semialdehyde[c]	Arginine and proline metabolism	More than 4 KJ/mol decrease	Precursor to other amino acids, involvement in energy metabolism, and immune cell activation[25].	Dendritic cells
CO2[m] + H2O[m] => H+[m] + HCO3-[m]	Arginine and proline metabolism	More than 6 KJ/mol increase	Important for ph regulation and Co2 transport for metabolic processes[51].	Dendritic cells
5-methylthioadenosine[c] + Pi[c] => adenine[c] + methylthioribose-1p[c]	Arginine and proline metabolism	More than 4 KJ/mol decrease	Precursor for methionine and nucleotides[52].	Dendritic cells
acetyl-CoA[m] + glutamate[m] => CoA[m] + H+[m] + N-acetyl-L-glutamate[m]	Arginine and proline metabolism	More than 5 KJ/mol increase	Precursor to other amino acids essential for cellular function.	Dendritic cells

H2O[c] + N-acetylornithine[c] <=> acetate[c] + ornithine[c]	Arginine and proline metabolism	More than 5 KJ/mol increase	Regulation of NO cycle and antimicrobial activity[47].	Dendritic cells
acetyl-CoA[c] + putrescine[c] => CoA[c] + H+[c] + N-acetylputrescine[c]	Arginine and proline metabolism	More than 4 KJ/mol decrease	Balance and proper functioning of polyamines[53].	Dendritic cells
AKG[c] + L-erythro-4-hydroxyglutamate[c] => glutamate[c] + 4-hydroxy-2-oxoglutarate[c]	Arginine and proline metabolism	More than 3 KJ/mol decrease	Maintenance of amino acid and nitrogen metabolism[37].	Dendritic cells
serine[c] + THF[c] <=> 5,10-methylene-THF[c] + glycine[c] + H2O[c]	Glycine, serine and threonine metabolism	More than 3 KJ/mol increase	Maintains cellular function and acts as a precursor for synthesizing proteins, nucleotides, and other biomolecules[52].	Dendritic cells
CoA[m] + L-2-amino-3-oxobutanoic acid[m] <=> acetyl-CoA[m] + glycine[m]	Glycine, serine and threonine metabolism	More than 3 KJ/mol decrease	Maintains cellular function and acts as a precursor for synthesizing proteins, nucleotides, and other biomolecules[42].	Dendritic cells
dehydroalanine[c] + H2O[c] => H+[c] + NH3[c] + pyruvate[c]	Glycine, serine and threonine metabolism	More than 3 KJ/mol decrease	Precursor for other amino acid metabolism[37].	Dendritic cells
serine[c] => dehydroalanine[c] + H2O[c]	Glycine, serine and threonine metabolism	More than 3 KJ/mol increase of + ΔG^o	Maintains cellular function and acts as a precursor for synthesizing	Dendritic cells

			proteins, nucleotides, and other biomolecules[42].	
1-pyrroline-5-carboxylate[m] + 2 H2O[m] + NAD+[m] => glutamate[m] + H+[m] + NADH[m]	Alanine, aspartate and glutamate metabolism	More than 3 KJ/mol decrease	Maintains cellular function and acts as a precursor for synthesizing proteins, nucleotides, and other biomolecules[42].	Dendritic cells
arginine[c] + H2O[c] => ornithine[c] + urea[c]	Arginine and proline metabolism	More than 3 KJ/mol decrease	Balance of NO cycle and ammonia levels[37].	Erythrocytes
H+[e] + ornithine[e] => CO2[e] + putrescine[e]	Arginine and proline metabolism	More than 5 KJ/mol increase	Balance of NO cycle and ammonia levels[47].	Erythrocytes
AKG[m] + L-erythro-4-hydroxyglutamate[m] => 4-hydroxy-2-oxoglutarate[m] + glutamate[m]	Arginine and proline metabolism	More than 5 KJ/mol increase	Precursor to other amino acids, involvement in energy metabolism, and immune cell activation[25].	Erythrocytes
4-hydroxy-2-oxoglutarate[m] => glyoxalate[m] + pyruvate[m]	Arginine and proline metabolism	More than 5 KJ/mol decrease	Precursor for many important amino acid metabolism[50].	Erythrocytes
H2O[c] + N1-acetylspermidine[c] + O2[c] => acetamidopropanal[c] + H2O2[c] + putrescine[c]	Arginine and proline metabolism	More than 3 KJ/mol decrease	Regulation of cellular growth, and maintenance of proper level of H2O2[54].	Erythrocytes

L-erythro-4-hydroxyglutamate[m] + OAA[m] => 4-hydroxy-2-oxoglutarate[m] + aspartate[m]<br>arginine[m] + H2O[m] => ornithine[m] + urea[m]	Arginine and proline metabolism	More than 4 KJ/mol increase	Link to glyoxylate metabolism and ROS control[32].	Erythrocytes
arginine[m] + H2O[m] => ornithine[m] + urea[m]	Arginine and proline metabolism	More than 3 KJ/mol decrease	Balance of NO and ammonia levels[37].	Erythrocytes
acetyl-CoA[c] + putrescine[c] => CoA[c] + H+[c] + N-acetylputrescine[c]	Arginine and proline metabolism	More than 3 KJ/mol increase	Regulation of polyamine levels[53].	Erythrocytes
3-phosphonooxypyruvate[c] + glutamate[c] <=> 3-phosphoserine[c] + AKG[c]<br>3-phosphoserine[c] + H2O[c] => Pi[c] + serine[c]	Glycine, serine and threonine metabolism	Almost 3 KJ/mol increase	Precursor to serine biosynthesis[55].	Erythrocytes
3-phosphoserine[c] + H2O[c] => Pi[c] + serine[c]	Glycine, serine and threonine metabolism	More than 3 KJ/mol increase	The precursor to serine metabolism and cellular proliferation mechanism[45].	Erythrocytes
serine[c] + THF[c] <=> 5,10-methylene-THF[c] + glycine[c] + H2O[c]	Glycine, serine and threonine metabolism	More than 4 KJ/mol decrease	Central to one-carbon metabolism is important for DNA synthesis and several amino acid metabolisms[55,56].	Erythrocytes
serine[c] => H+[c] + NH3[c] + pyruvate[c]	Glycine, serine and threonine metabolism	More than 3	Balance of proper ammonia level[s37].	Erythrocytes

		KJ/mol decrease		
pyruvate[c] + serine[c] => alanine[c] + hydroxypyruvate[c]	Glycine, serine and threonine metabolism	More than 3 KJ/mol increase in + ΔGº	Connection to several amino acid metabolism and energy metabolism[49]	Erythrocytes
serine[c] => dehydroalanine[c] + H2O[c]	Glycine, serine and threonine metabolism	More than 3 KJ/mol decrease	Important for modification of proteins and precursors to other amino acids as needed[56].	Erythrocytes
pyruvate[x] + serine[x] => alanine[x] + hydroxypyruvate[x]	Glycine, serine and threonine metabolism	More than 3 KJ/mol increase in + ΔGº	The interrelationship between amino acid and carbohydrate metabolism[57].	Erythrocytes

Supplementary Table 5: Table showing results from Shadow Price Analysis which allows the identification of constraints that either positively and negatively impact the objective function (in our case that is minimum driving force of the pathway). The Positive Shadow Price indicates, that particular reaction is limiting the objective function in positive direction and negative value indicates impact on negative direction. From our analysis, we identify multiple reactions originating from several key pathways such as Arginine and proline metabolism, Amino sugar and nucleotide sugar metabolism, and glycolysis/gluconeogenesis. The negative shadow prices indicate that these are the steps currently defining the minimum driving force. Relaxing their thermodynamic constraints (e.g., via metabolite concentration adjustments) would directly increase the overall MDF.

**Reaction**	**Shadow Price**	**Equation**	**Pathway**
MAR03837	-0.14286	1-pyrroline-5-carboxylate[m] + 2 H+[m] + NADH[m] => NAD+[m] + proline[m]	Arginine and proline metabolism
MAR04779	-0.28571	NAD+[m] + trans-4-hydroxy-L-proline[m] => 2 H+[m] + L-1-pyrroline-3-hydroxy-5-carboxylate[m] + NADH[m]	Arginine and proline metabolism
MAR04784	-0.14286	2 H2O[m] + L-1-pyrroline-3-hydroxy-5-carboxylate[m] + NAD+[m] => H+[m] + L-erythro-4-hydroxyglutamate[m] + NADH[m]	Arginine and proline metabolism
MAR08097	-0.28571	2 H+[m] + L-erythro-4-hydroxyglutamate[m] + NADH[m] <=> H2O[m] + L-4-hydroxyglutamate semialdehyde[m] + NAD+[m]	Arginine and proline metabolism
MAR08611	-0.14286	FAD[m] + proline[m] => 1-pyrroline-5-carboxylate[m] + FADH2[m] + H+[m]	Arginine and proline metabolism
MAR04529	-1	H2O[c] + N-acetylmannosamine-6-phosphate[c] + PEP[c] => N-acetylneuraminate-9-phosphate[c] + Pi[c]	Amino sugar and nucleotide sugar metabolism

MAR03905	-0.5	ethanol[c] + NAD+[c] => acetaldehyde[c] + H+[c] + NADH[c]	Glycolysis / Gluconeogenesis
MAR04388	-0.5	H+[c] + NADH[c] + pyruvate[c] <=> L-lactate[c] + NAD+[c]	Glycolysis / Gluconeogenesis
MAR04379	-0.33333	ATP[c] + fructose-6-phosphate[c] => ADP[c] + fructose-1,6-bisphosphate[c] + H+[c]	Glycolysis / Gluconeogenesis
MAR04358	-0.33333	ADP[c] + H+[c] + PEP[c] => ATP[c] + pyruvate[c]	Glycolysis / Gluconeogenesis
MAR04363	-0.33333	2-phospho-D-glycerate[c] <=> H2O[c] + PEP[c]	Glycolysis / Gluconeogenesis
MAR01080	-0.5	H2O[c] + leukotriene A4[c] => leukotriene B4[c]	Leukotriene metabolism
MAR01100	-0.5	leukotriene B4[c] + NAD+[c] => 12-keto-LTB4[c] + H+[c] + NADH[c]	Leukotriene metabolism
MAR06912	-0.36364	H2O[m] + PPi[m] => H+[m] + 2 Pi[m]	Oxidative phosphorylation
MAR06916	-0.36364	ADP[m] + Pi[m] + 3 H+[i] => ATP[m] + 2 H+[m] + H2O[m]	Oxidative phosphorylation
MAR06921	-0.27273	5 H+[m] + NADH[m] + ubiquinone[m] => NAD+[m] + ubiquinol[m] + 4 H+[i]	Oxidative phosphorylation
MAR04398	-0.25	2-deoxy-D-ribose-5-phosphate[c] <=> acetaldehyde[c] + D-glyceraldehyde 3-phosphate[c]	Pentose phosphate pathway
MAR04565	-0.25	D-glyceraldehyde 3-phosphate[c] + sedoheptulose-7-phosphate[c] <=> erythrose-4-phosphate[c] + fructose-6-phosphate[c]	Pentose phosphate pathway
MAR04567	-0.25	ATP[c] + sedoheptulose-7-phosphate[c] => ADP[c] + H+[c] +	Pentose phosphate pathway

		sedoheptulose-1,7-bisphosphate[c]	
MAR08074	-0.25	ATP[c] + deoxyribose[c] => 2-deoxy-D-ribose-5-phosphate[c] + ADP[c] + H+[c]	Pentose phosphate pathway
MAR03859	-1	D-lactate[c] + 2 ferricytochrome C[m] => 2 ferrocytochrome C[m] + 2 H+[c] + pyruvate[c]	Pyruvate metabolism

Supplementary Table 6: Shadow price analysis highlights metabolites that function as bottlenecks and directly influence the pathway's overall MDF. Several metabolites originating from different metabolic routes were identified, indicating that changes in their concentrations would strongly affect the MDF. In particular, our analysis revealed multiple metabolites with positive shadow prices, showing that the MDF is especially sensitive to thermodynamic constraints within arginine and proline metabolism, amino sugar and nucleotide sugar metabolism, and glycolysis/gluconeogenesis. The concentration bounds of these metabolites therefore represent the main thermodynamic limitations of the pathway under the tested conditions and serve as high-priority targets for metabolic or thermodynamic engineering aimed at increasing pathway driving forces.

**Metabolite ID**	**Metabolite Name**	**Shadow Price**
MAM01862m	fumarate	2.57873
MAM01803m	FADH2	2.57873
MAM02883c	sedoheptulose-1,7-bisphosphate	0.64468
MAM01672c	deoxyribose	0.64468
MAM03103m	ubiquinone	0.70329
MAM02039m	H+	0.70329
MAM02759m	PPi	0.93772
MAM01285m	ADP	0.93772
MAM02553m	NADH	0.70329
MAM02040c	H2O	1.28936
MAM02362c	leukotriene A4	1.28936
MAM02552c	NAD+	1.28936
MAM01845c	fructose-6-phosphate	0.87066
MAM00674c	2-phospho-D-glycerate	0.87066
MAM01796c	ethanol	1.28936
MAM02819c	pyruvate	1.28936
MAM02040c	H2O	2.57873
MAM02539c	N-acetylmannosamine-6-phosphate	2.57873

MAM02696c	PEP	2.57873
MAM03037m	trans-4-hydroxy-L-proline	0.73678
MAM02358m	L-erythro-4-hydroxyglutamate	0.36839
MAM01802m	FAD	0.36839
MAM00913c	3-phospho-D-glycerate	1.28929
MAM02475c	methylglyoxal	1.28929
